# Secondary neoplasm to non-hodgkin lymphoma treatment manifesting as a cancer of unknown primary: the first case in literature

**DOI:** 10.1097/MS9.0000000000001881

**Published:** 2024-03-04

**Authors:** George Bashour, Nina Kheyrbek, Ali Dway, Elias Salloum, Michael Georgeos, Zuheir Alshehabi

**Affiliations:** a Cancer Research Center; b Faculty of Medicine; Departments of c Oncology; d Pathology, Tishreen University Hospital, Latakia; e Faculty of Medicine, Al-Andalus University, Tartus, Syria

**Keywords:** cancer of unknown primary, immunohistochemistry, secondary neoplasm

## Abstract

**Introduction:**

Cancer of unknown primary (CUP) is a tumour metastasis with no detectable primary origin. A secondary neoplasm (SN) is defined as a tumour secondary to a prior tumour treatment and has no histological relation to that primary tumour.

**Case presentation:**

The authors report a case of a 72-year-old female patient who presented with back pain and had a history of non-Hodgkin lymphoma (NHL) treated with RCHOP 12 years ago. MRI showed a compression fracture in T5 and T7 vertebrae, while the PET/computed tomography (CT) only showed hypermetabolic lytic bone lesions in these vertebrae. Pathological examination of a biopsy of these lesions suggested metastatic breast cancer, but the mammography was normal. The above clinical description indicates that our case is a SN to RCHOP treatment manifested as a cancer of unknown origin.

**Discussion:**

CUP is diagnosed when all screening procedures fail to find the original tumour. On the other hand, the literature showed that RCHOP treatment of non-Hodgkin lymphoma has a 0.68% chance of causing a SN. After an extensive literature search, we found that our case, which has the combination of both CUP and SN, is the first documented case.

**Conclusion:**

This case suggests that cancer patients who received chemical or radiological treatment should be screened more carefully on the long term as it is possible to developed secondary neoplasms without a primary tumour in areas difficult to diagnose with traditional screening tools.

## Introduction

Highlights

- Cancer of unknown primary is metastasis with no detectable primary tumour.
- Secondary neoplasm is a new tumour induced by chemo and radiotherapy.
- We report the first case in literature of a secondary neoplasm manifesting as cancer of unknown primary.
- Our case highlights the need for further research on secondary neoplasm to chemotherapy and radiotherapy.
- Cancer patients should be screened more carefully on the long term after chemotherapy and radiotherapy.

Cancer of unknown primary (CUP) is defined as a cancer for which only metastasis can be found at the time of diagnosis with no detectable primary tumour despite thorough investigations^1^.

CUP accounts for 2–5% of cancers diagnosed worldwide and is an early and aggressive metastasis, and although it is rare it counts as the fourth most common cause of cancer deaths^2^.

In the available literature, a higher cure rate has been reported in patients with known underlying disease than in patients with CUP (77.2% vs. 51.1%)^1^.

Secondary malignancies or secondary neoplasm (SN) differ from metastasis in being caused by radiotherapy or chemotherapy of the prior tumour and different from recurrence as it has no histological relation to the first cancer treated^3^. Radiotherapy is the most important risk factor for SN^4^. Also, higher cure rates in cancer patients help in longevity, which is found to increase the risk of secondary neoplasms^5^.

After a thorough search of medical literature, we have found no cases of SN manifesting as a CUP syndrome.

Here we present the first case, to our knowledge, in the literature of secondary neoplasm manifesting as a CUP syndrome.

### Presentation

A 72-year-old female patient, non-smoker, non-alcoholic, presented to our clinic with back pain. The clinical examination showed no significant findings. She had a history of treated non-Hodgkin lymphoma of the tonsils 12 years ago with RCHOP with local radiotherapy. The patient also had no prior exposure to contraceptives nor any family history of any type of cancer. The lab report was normal except for a rise in ESR and platelet count. The patient was found to be a type 2 diabetic with a fasting glucose of 176 mg/dl.

**Figure 1:**
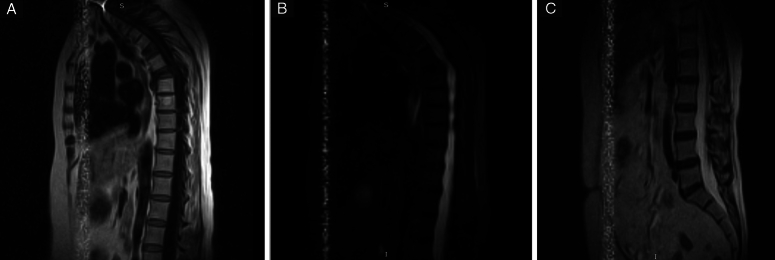
Magnetic resonance imaging shows: T1, compression fracture in T5 and T7 with backward displacement of the vertebral body in (A). T2, compression of the thoracic spinal cord (B). T1, no changes or injury in the lumbar area (C).

MRI showed a compression fracture in T7 with backward displacement of the vertebral body causing pressure on the spinal cord. Also, there was spondylolisthesis of T6/T7 causing localized kyphosis and compression fracture in T5 with a left vertebral pedicle mass (Fig. 1).

**Figure 2:**
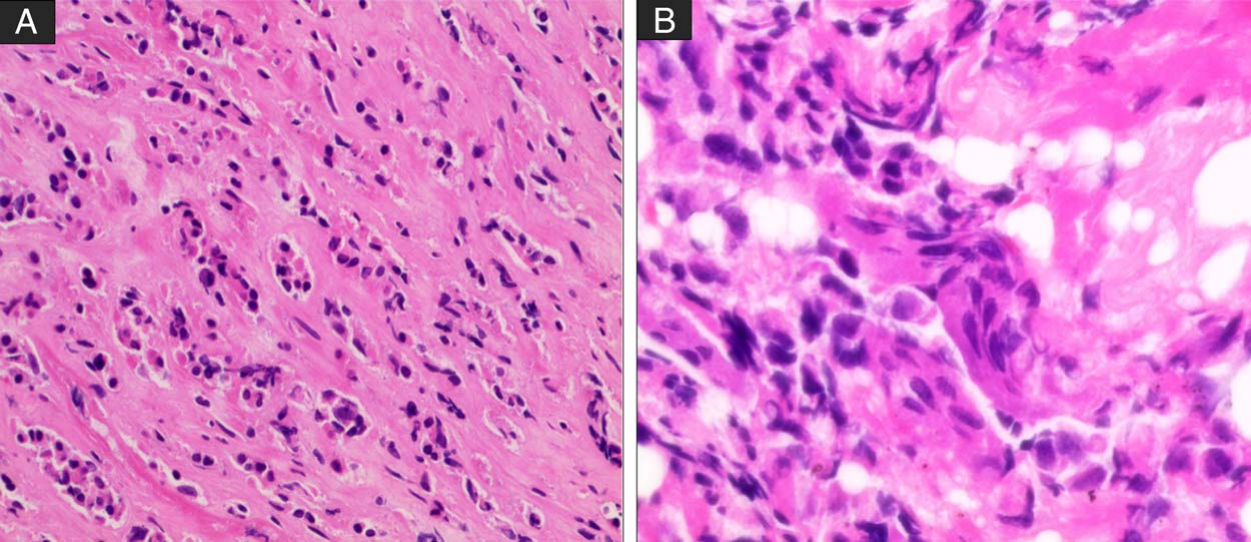
Histopathology of the tumour: Low power view shows infiltration of bone tissue by atypical neoplastic epithelial cells, occurring in cords, glandular/ductal, and isolated forms [hematoxylin and eosin (H&E), 100×] (A), The neoplastic cells dissecting between necrotic tissue, showing hyperchromatic nuclei, scanty basophilic cytoplasm, and occasional mitotic figures (H&E, 200×) (B).

**Figure 3:**
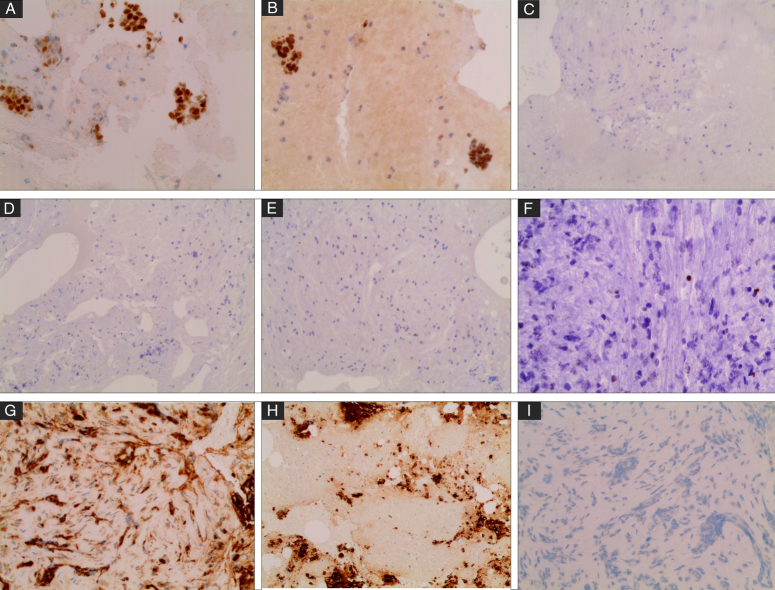
Immunohistochemistry of the tumour biopsy: CK-positive (A), CK7-positive (B), ER-positive (C), PR-negative (D), GATA-3-positive (E), P-53-negative (F), TTF-1-negative (I), WT-1-negative (G), PAX-8-negative (H).

A PET/computed tomography (CT) scan showed multiple hypermetabolic predominantly lytic bone lesions in both T5 and T7 with no other findings. Further evaluation by vertebral biopsy was consistent with metastatic carcinoma. Immune stains showed positivity for keratin expression (CK, CK7), ER, and GATA-3 while being negative for PR, HER-2 neu, P53, PAX-8, WT-1, and TTF-1(Figs. 2 and 3). The above findings suggested the diagnosis of metastatic breast or uterine carcinoma.

**Figure 4:**
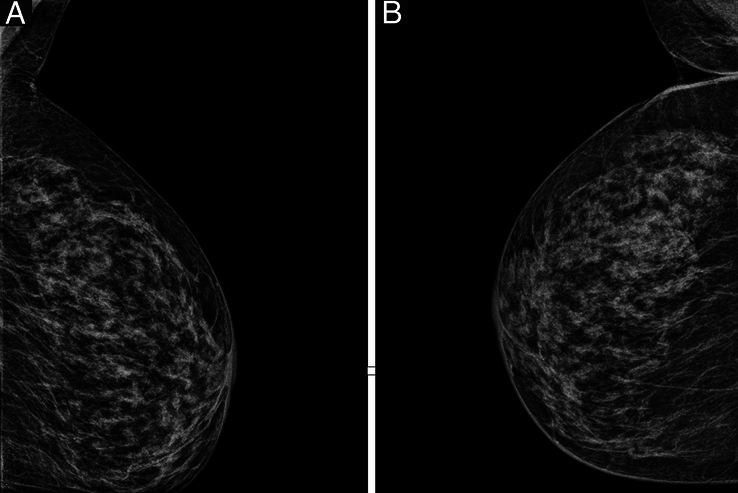
Mammography of both left (A) and right (B), breasts show no pathological changes BI-RADS stage: 2/6.

Mammography of both breasts was normal and showed no suspicious calcifications or density (Fig. 4). Ultrasound (U/S) of the pelvic region was normal. The patient received radiotherapy: 30 Gray in 10 fractions. Radiotherapy was applied at the level of T4–T8 with regular image guidance via cone beam CT (CBCT). The patient was put on a course of Palbociclib (Ibrance 150 mg) and Letrozole 2.5 mg. The patient reported symptom improvement after 2 months, but she neglected her treatment after the 6th month as she improved significantly.

## Discussion

The clinical definition of CUP also referred to as malignancy of unknown origin (MUO) syndrome refers to patients who develop histologically proven metastatic cancer with no clear indication of the original tumour. Our patient was examined thoroughly to identify the potential primary tumour following the pathology report with no result. Her mammography was normal, and her PET/CT scan and MRI showed only vertebral metastasis in T5 and T7. Although pathology suggested breast or uterine carcinoma, all these screening procedures failed to detect a primary tumour. Thus, we identify our case as CUP syndrome. The symptoms of CUP are determined by the organ involved, but most patients are asymptomatic. the lymph nodes make almost 40% of affected organs, followed by the liver, the lungs, and the bones making ~35%. The least affected organs are the pleura, the peritoneum, the central nervous system (CNS), and the adrenal glands^6^. Under routine staining, about 50% of CUP cases can be classified as well-to-moderately differentiated adenocarcinomas, 30% as poorly or undifferentiated adenocarcinomas, 15% as squamous cell carcinomas, and 5% as undifferentiated neoplasms, even though the tissue of origin is not readily identified^7^. IHC is a crucial tool used to further assist in diagnosing and locating the tumour^7^. Epithelial, lymphoid, and melanocyte antigens are frequently included in the first panel used to identify undifferentiated neoplasms or cells with unknown lineages^7^. CK7 and CK20 included in the second panel if the cells are thought to be of epithelial lineage^7^ In recent years, IHC has also been used to identify the expression of more specific cancer-related proteins and treatment-response predictors^7^. Our case showed this modern usage of IHC in suggesting the treatment plan as highly expressed ER tends to have a better response when treated with aromatase inhibitors (AI)^8^. The patient got AI treatment along Palbociclib. This combination has a better prognosis as proved by Brufsky *et al*.^9^, who found that this combination had increased tumour response compared with those treated with letrozole alone. In a study by CHO and colleagues, 1607 patients who received RCHOP, 11 (0.68%) patients developed secondary primary cancer. Our patient had a history of tonsils NHL which was treated with RCHOP regimen, which makes this new tumour a SN. Thus, our case is a SN manifesting as a CUP syndrome.^10^

Finally, the processes that cause CUP syndrome and the development of this orphan disease are not fully understood^1^. The unfavourable survival expectancy of 12 months^1^ makes the matter of understanding and treating CUP more urgent to increase patients’ survival. Also, the increasing rate of SN with more availability of cancer treatment worldwide makes long-term management a more complex mission.

## Conclusion

Here we report a case of vertebral metastasis of a pathologically suggested breast tumour, after thorough examination with PET/CT, MRI, and mammography, no primary tumour was found and with the patient’s history of NHL treated with RCHOP and the histological difference, this is in correlation with secondary neoplasm manifesting as a cancer of unknown primary. To our knowledge, this is the first case in literature to combine these two occurrences. Our case suggests that cancer patients who received chemical or radiological treatment should be screened more carefully on the long term as it is possible to developed secondary neoplasms without a primary tumour in areas difficult to diagnose with traditional screening tools.

## Ethical approval

Not applicable.

## Consent for publication

Written informed consent was obtained from the patient for publication of this case report and any accompanying images. A copy of the written consent is available for review by the Editor-in-Chief of this journal upon request.

## Source of funding

Not applicable.

## Authors contribution

All authors contributed to this manuscript. G.B., N.K., A.D., E.S.: writing—original draft, reviewing and editing. G.B.: supervision. M.G., Z.A.: reviewing and editing.

## Conflicts of interest disclosure

The authors declare no conflict of interest.

## Research registration unique identifying number (UIN)

Not applicable

## Guarantor

Zuheir Alshehabi.

## Data availability statement

The data are available on request.

## Provenance and peer review

Not commissioned, externally peer-reviewed.
